# Underfunded, Overperforming: Endometrial cancer leads basic and translational science publications per NCI dollar

**DOI:** 10.1016/j.gore.2026.102244

**Published:** 2026-11-03

**Authors:** Michael T. Richardson, Anne Gallivan, Lily Zello, Miles Morrow, Daniel S. Kapp, John K. Chan, Melissa Herbst-Kralovetz, Dana M. Chase

**Affiliations:** a University of California Los Angeles, Los Angeles, CA, USA; b University of California, Santa Barbara, CA, USA; c Department of Radiation Oncology, Stanford University School of Medicine, Stanford, CA, USA; d California Pacific/Palo Alto Medical Foundation, Sutter Health, San Francisco, CA, USA; e University of Arizona, Phoenix, AZ, USA

**Keywords:** Healthcare disparities, Research funding, Bibliometrics, Genital neoplasms (female), Ovarian neoplasms

## Abstract

**Objective:**

To compare relative research funding and basic and translational science publications for gynecologic cancers vs. other common cancers, normalized to incidence and mortality rates.

**Methods:**

SEER data were used to investigate incidence and deaths in the US from 2004 to 2024. Research funding was derived from National Cancer Institute (NCI) data. Google Scholar was utilized to identify oncology and medicine related journals with the highest H-indices. All abstracts on PubMed were reviewed and number of basic and translational science articles manually calculated.

**Results:**

In 2024, new diagnoses of breast, prostate, lung, and colorectal cancers totaled 313,510, 299,010, 234,580, and 152,810, respectively. Endometrial, ovarian, and cervical cancers accounted for 67,880, 19,680, and 13,820, respectively.

NCI funding in 2023 was approximately $542.0 million for breast, $258.6 million for prostate, $435.0 million for lung, and $238.6 million for colorectal cancers, compared to $20.0 million, $132.0 million, and $20.7 million for endometrial, ovarian, and cervical cancers, respectively.

Across the highest impact journals from 2004 to 2024, 24,169 basic/translational science publications were found: 8859 (breast), 4161 (prostate), 3537 (lung), 4321 (colorectal), 412 (endometrial), 2231 (ovarian), and 648 (cervical). When adjusted for incidence, ovarian cancer had the highest number of publications, at 220.9 publications per 100,000 cases. When adjusted for funding, endometrial cancer had the highest number of publications, at 23.1 publications per $1 million average annual NCI funding.

**Conclusion:**

Funding relative to disease burden varies considerably among gynecologic cancers. Endometrial cancer receives the lowest NCI funding of any cancer studied when adjusted by incidence and mortality. Despite this, endometrial cancer produced the most basic and translational science publications per dollar.

## Highlights

• NCI funding for endometrial cancer trails other major cancer types.
• Endometrial cancer receives the lowest NCI funding relative to incidence/mortality.
• Endometrial cancer had the most publications per NCI dollar.

## 1 Introduction

Despite advances in the prevention of HPV-driven cancers and novel life-prolonging therapeutics, the burden of gynecologic cancers continues to rise (Lei et al., 2020; Liao et al., 2022; Wilson et al., 2024). Worldwide, new cases of gynecologic malignancies are expected to increase by nearly 50% by 2050 (Zhu et al., 2024). In the United States, uterine cancer incidence and mortality are rapidly increasing (Wright et al., 2025). Addressing this crisis requires a sustained investment in scientific discovery, but funding for gynecologic cancers remains disproportionately low. A study by Spencer et al. demonstrated that when adjusted for lethality, funding from the National Cancer Institute (NCI) for prostate cancer research was nearly twenty-times higher than for ovarian cancer, with disparities even greater for cervical and endometrial cancer (Spencer et al., 2019). Disparate federal funding is particularly concerning as federal grants face increasing risk of termination (Liu et al., 2025).

This disproportionate funding may be affecting scientific output. A study by Sinha et al. found that disparities in funding are associated with decreased publication rates for gynecologic cancers in the highest impact factor journals (Sinha et al., 2023). However, their study focused on publication rates in general, without characterizing each publication. The degree to which these funding disparities affect basic and translational science productivity specifically remains unknown.

Given the central role of governmental investment in early research development, understanding how these funding disparities affect basic and translational science output is essential (Galkina Cleary et al., 2023; Galkina Cleary et al., 2018; Azoulay et al., 2019). For this reason, we sought to determine the extent to which this disproportionately low research funding for gynecologic malignancies is reflected in basic and translational science publication output.

## 2 Methods

Rates of cancer diagnoses, deaths, incidence, mortality, NCI funding, and publication rates were compared between the most common gynecologic malignancies (endometrial, ovarian, and cervical) and the other most common malignancies in the United States (breast, prostate, lung, colorectal). These latter cancer sites were selected as they represent the four most common cancer diagnoses, and approximately half of all cancers in the United States (Siegel et al., 2024). The combination of ovarian, cervical, and endometrial cancers was collectively described as “Gyn” for the purpose of this analysis, as these together represent nearly 90% of incident gynecologic malignancies (Siegel et al., 2026).

Total cancer diagnoses and deaths were derived from the American Cancer Society annual publications from 2004 and 2024 (Siegel et al., 2024; Jemal et al., 2004). Incidence and mortality data were derived from the Surveillance, Epidemiology, and End Results (SEER) program and reported per 100,000 (National Cancer Institute, n.d.). Federal funding data were derived from the NCI Budget Fact Book (National Cancer Institute Budget Fact Book, 2026). Data from these sources were reported up to their most recent year published (2022 for incidence data, 2023 for NCI funding, and 2023 for mortality). Average annual NCI funding was then compared against most recent incidence and mortality data.

Incidence, mortality, and NCI funding were then compared to basic and translational science publication rates and trends. To derive these publication data, the PubMed database was queried for English language original research articles published between 2004 and 2024 that focused on basic and/or translational science. PubMed search terms are displayed in Supplementary Data. Searches were limited to very high impact journals, as defined by Google Scholar's 2024H-index rankings in the category *Health and Medical Sciences* and subcategory *Oncology*. The twenty highest-ranked journals from each list were included along with selected additional general science journals (Supplementary Data).

Article titles, abstracts, and manuscripts were then manually reviewed by investigators (MR, AG, LZ, MM) to determine if they met criteria for inclusion as a basic or translational science article. Each search was performed in duplicate and discordant classifications adjudicated by MR. Only studies that focused on a particular cancer were included. For example, a study encompassing multiple tumor types that included cervical cancer among others would be excluded. Clinical trials, imaging studies, meta-analyses, systematic reviews, and clinical investigations were also excluded, as were commentaries, corrections, and erratum. Clinical trials were excluded, as this study focused specifically on basic and translational science output. Additionally, enrollment in cancer clinical trials is more frequently attributable to industry than to federal sponsorship, and their inclusion may therefore less accurately reflect the effects of federal research investment (Unger et al., 2024). As this study included only publicly available data that did not contain identifiable patient data, the study was deemed exempt from IRB oversight.

Comparisons across cancer types were conducted using descriptive statistical methods and ANOVA testing. Statistical analyses were performed using SPSS (IBM Corp., Armonk, NY) and graphical representation through Microsoft Excel 365 (Microsoft Corporation, (2024)).

## 3 Results

In 2024, the most common cancers in the United States (excluding basal and squamous cell skin cancers) were breast (313,510 cases), prostate (299,010), lung (234,580), and colorectal (152,810, Table 1). Gynecologic malignancies consisted of endometrial (67,880 cases), ovary (19,680) and cervix (13,820). Cases with the highest estimated deaths were lung (125,070), colorectal (53,010), breast (42,780), and prostate (35,250). Among gynecologic cancers, deaths were highest from endometrial (13,250), ovarian (12,740), and cervical cancers (4360).

**Table 1:** Trends in diagnoses, deaths, incidence, and mortality.

	Breast	Prostate	Lung	Colorectal	Endometrial	Ovary	Cervix	Gyn
New Cases 2004 (n)	217,440	230,110	173,770	146,940	40,320	25,580	10,520	76,420
New Cases 2024 (n)	313,510	299,010	234,580	152,810	67,880	19,680	13,820	101,380
New Cases Change	+44.2%	+29.9%	+34.9%	+3.9%	+68.4%	−23.1%	+31.4%	+32.7%
Deaths 2004 (n)	40,580	29,900	160,440	56,730	7090	16,090	3900	27,080
Deaths 2024 (n)	42,780	35,250	125,070	53,010	13,250	12,740	4360	30,350
Death Change	+5.4%	+17.9%	−22.0%	−6.6%	+86.9%	−20.8%	+11.8%	+12.1%
Incidence 2004	124.6	161.4	56.0	48.1	23.6	13.2	7.8	44.6
Incidence 2022	135.3	120.4	38.4	35.4	28.8	10.1	6.9	45.8
Incidence Change	+8.6%	−25.4%	−31.4%	−26.4%	+22.0%	−23.5%	−11.5%	+2.7%
Mortality 2004	24.5	26.6	53.3	18.1	4.1	8.8	2.4	15.3
Mortality 2023	18.9	18.7	29.4	12.7	5.6	5.5	2.1	13.2
Mortality Change	−22.9%	−29.7%	−44.8%	−29.8%	+36.6%	−37.5%	−12.5%	−13.7%

Incidence and Mortality Reported as observed rate, per 100,000.

Gyn: Combination of endometrial, ovary, and cervix cancers.

When adjusted for population size, cancer incidence increased most for endometrial cancer (from 23.6 to 28.8 cases per 100,000, 22.0% increase) and breast cancer (124.6 to 135.3, 8.6% increase), while decreasing for all other cancers (Table 1). Nearly all listed cancers had a decrease in mortality as well. For example, lung cancer mortality decreased by 44.8% (53.3 to 29.4), ovarian by 37.5% (8.8 to 5.5), colorectal by 29.8% (18.1 to 12.7), prostate by 29.7% (26.6 to 18.7), and cervix by 12.5% (2.4 to 2.1). In contrast, mortality of endometrial cancer increased by 36.6% (4.1 to 5.6 per 100,000).

In 2023, NCI funding was highest for breast ($542.0 million), lung ($435.0 million), prostate ($258.6 million), and colorectal ($238.6 million) cancers. Funding for each cancer individually was greater than all Gyn cancers combined ($172.7 million, comprised of $20.0 million for endometrial, $132.0 million for ovary, and $20.7 million for cervix, Table 2). While total NCI funding increased by 52.9% from 2004 to 2023 ($4.7 billion to $7.2 billion), overall funding for gynecologic malignancies decreased over the study period, from $205.6 million to $172.7 million (16.0% decrease). Cervical and endometrial cancers were most adversely affected by funding decreases – cervical cancer funding decreased by 73.8% and endometrial by 25.9%. Endometrial cancer received the lowest average NCI funding relative to disease burden, with $0.62 million per unit of incidence (cases per 100,000) and $3.19 million per unit of mortality (deaths per 100,000, *p* < 0.001, Fig. 1). Relative to incidence, this represented 7.5% of the funding received by lung cancer.

**Table 2:** NCI funding trends by cancer type and comparison to incidence and mortality.

	Breast	Prostate	Lung	Colorectal	Endometrial	Ovary	Cervix	Gyn	*p*-value
2004 NCI funding	$566.2 m	$308.5 m	$276.5 m	$262.0 m	$27.0 m	$99.6 m	$79.0 m	$205.6 m	
2023 NCI funding	$542.0 m	$258.6 m	$435.0 m	$238.6 m	$20.0 m	$132.0 m	$20.7 m	$172.7 m	
Funding change (%)	−4.3%	−16.2%	+57.3%	−8.9%	−25.9%	+32.5%	−73.8%	−16.0%	
Average NCI Funding⁎	$567.0 m	$265.5 m	$317.2 m	$244.5 m	$17.9 m	$109.5 m	$71.7 m	$199.1 m	
Funding to incidence ratio	4.2	2.2	8.3	6.9	0.6	10.8	10.4	4.4	<0.001
Funding to mortality ratio	30.0	14.2	10.8	19.3	3.2	19.9	34.2	15.1	<0.001

All funding data reported in millions of US dollars.

All funding ratio data reported in average millions of dollars NCI funding, per observed rate of incident cancers or mortality (per 100,000), for most recent years available (2022 for incidence, and 2023 for mortality).

Gyn: Combination of endometrial, ovary, and cervix cancers.

⁎ Average annual NCI funding calculated as the mean of annual NCI funding from 2004 to 2023.

**Fig. 1:**
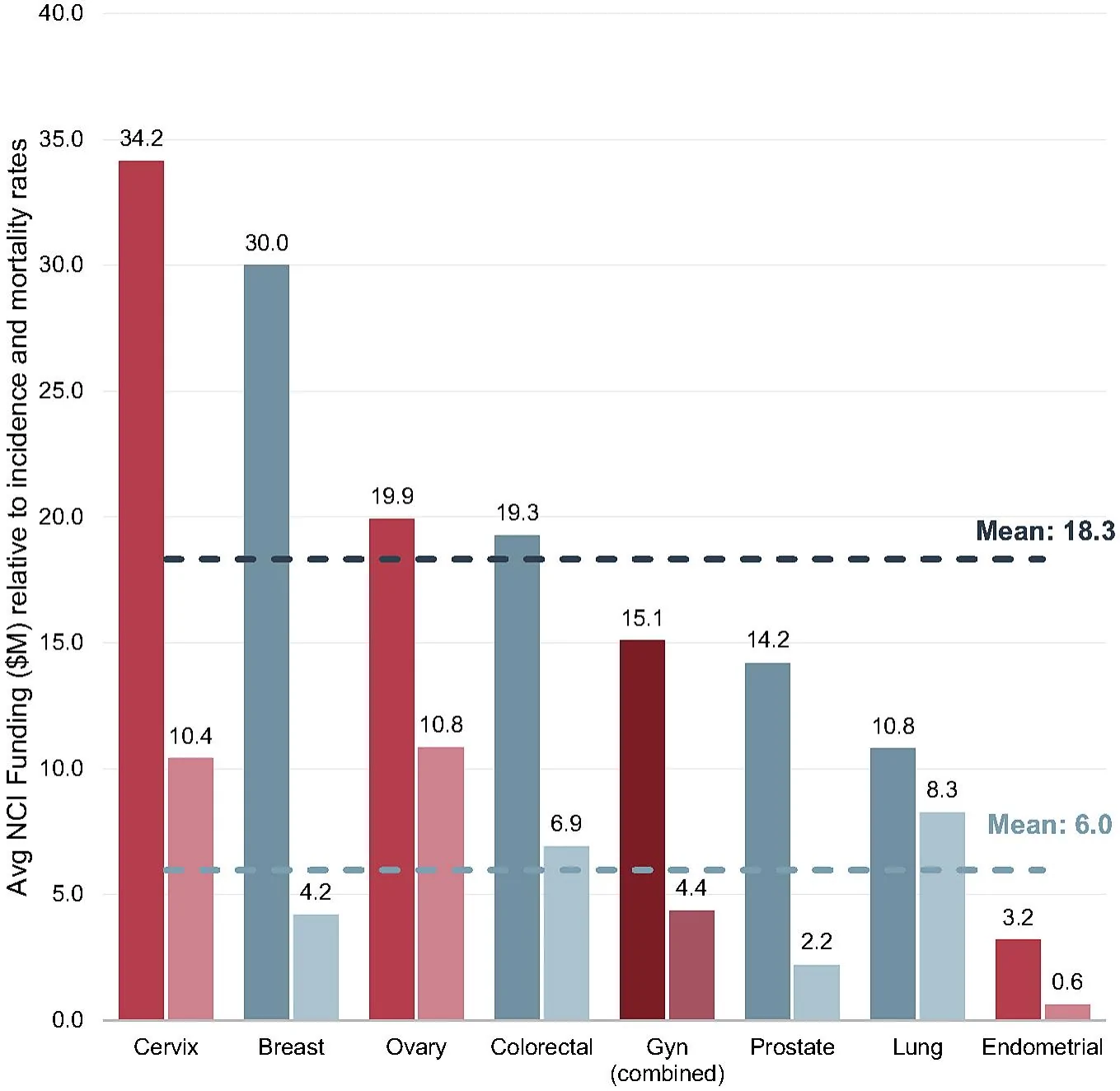
Average annual NCI funding relative to incidence and mortality rates, by cancer type. All funding ratio data reported in average millions of dollars NCI funding, per observed rate of incident cancers or mortality (per 100,000), for most recent years available (2022 for incidence, and 2023 for mortality). Dark bars represent funding to mortality ratio. Light bars represent funding to incidence ratios. Colors represent cancer category (red: gynecologic cancer, blue: comparison cancers). (For interpretation of the references to color in this figure legend, the reader is referred to the web version of this article.)

We next examined whether these funding disparities were reflected in basic and translational science publication output. From the top journals ranked by H-index, there were 24,169 published basic or translational science articles that focused on breast, prostate, lung, colorectal, or gyn cancers identified between 2004 and 2024 (Table 3, Fig. 2). The greatest number of publications focused on breast (8859), prostate (4161), lung (3537), and colorectal (4321) cancers. Gynecologic cancers accounted for 3291 total basic and translational science publications (412 for endometrial cancers, 2231 for ovary, and 648 for cervix).

**Table 3:** Basic and translational science publication data.

	Breast	Prostate	Lung	Colorectal	Endometrial	Ovary	Cervix	Gyn	p-value
Number Publications	8859	4161	3537	4321	412	2231	648	3291	
Publications 2004–2009	1274	898	437	412	50	329	59	438	
Publications 2010–2017	3535	1523	1161	1207	102	777	214	1093	
Publications 2018–2024	4050	1740	1939	2702	260	1125	375	1760	
Change*	+172%	+66%	+280%	+462%	+346%	+193%	+445%	+244%	
Publications to incidence	65.5	34.6	92.1	122.1	14.3	220.9	93.9	71.9	<0.001
Publications to mortality	468.7	222.5	120.3	340.2	73.6	405.6	308.6	249.3	<0.001
Publications to funding	15.6	15.7	11.2	17.7	23.1	20.4	9.0	16.5	<0.001

Publication change calculated by average publication per year from 2004 to 2009 compared to average publication per year 2018–2024.

Publication to incidence and mortality ratios defined as number of total publications over the study period per observed rate of incident cancers or mortality (per 100,000) for most recent years available (2022 for incidence, and 2023 for mortality).

Publication to funding defined as number of publications per million USD by average annual NCI funding (2004 to 2023).

Gyn: Combination of endometrial, ovary, and cervix cancers.

**Fig. 2:**
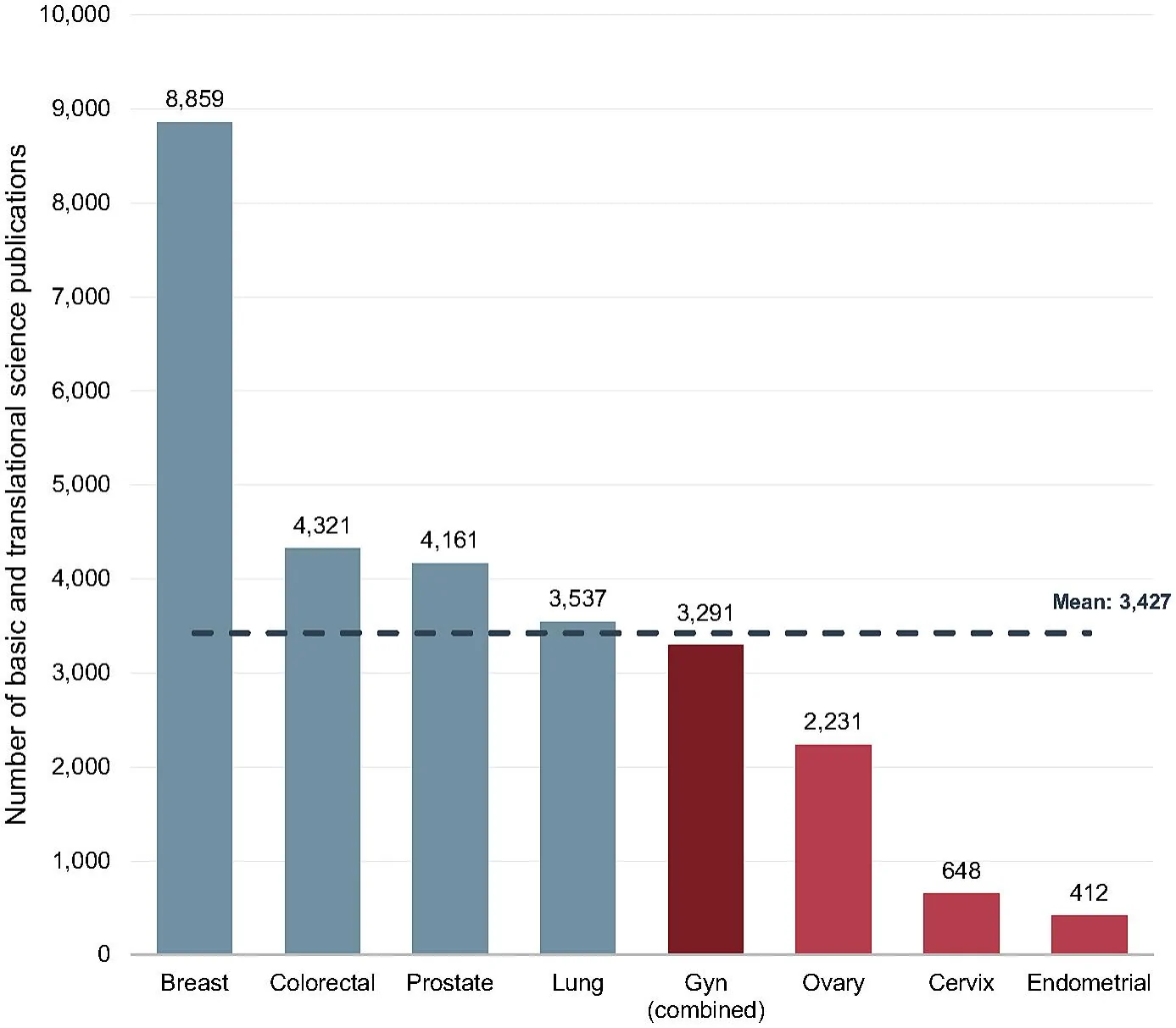
Total number of basic and translational science publications by cancer site. Colors represent cancer category (red: gynecologic cancer, blue: comparison cancers). (For interpretation of the references to color in this figure legend, the reader is referred to the web version of this article.)

When adjusted for incidence, ovarian cancer had the highest number of publications, at 220.9 publications per 100,000 cases (*p* < 0.001, Fig. 3A), while endometrial cancer had the lowest (14.3). When adjusted for mortality, breast cancer had the highest number of publications, at 468.7 publications per 100,000 deaths (*p* < 0.001, Fig. 3B), while endometrial cancer had the lowest (73.6). When adjusted for funding, endometrial cancer had the highest number of publications (23.1) per $1 million USD NCI funding, followed by ovarian cancer at 20.4 (*p* < 0.001, Fig. 3C), while cervix cancer had the lowest (9.0).

**Fig. 3:**
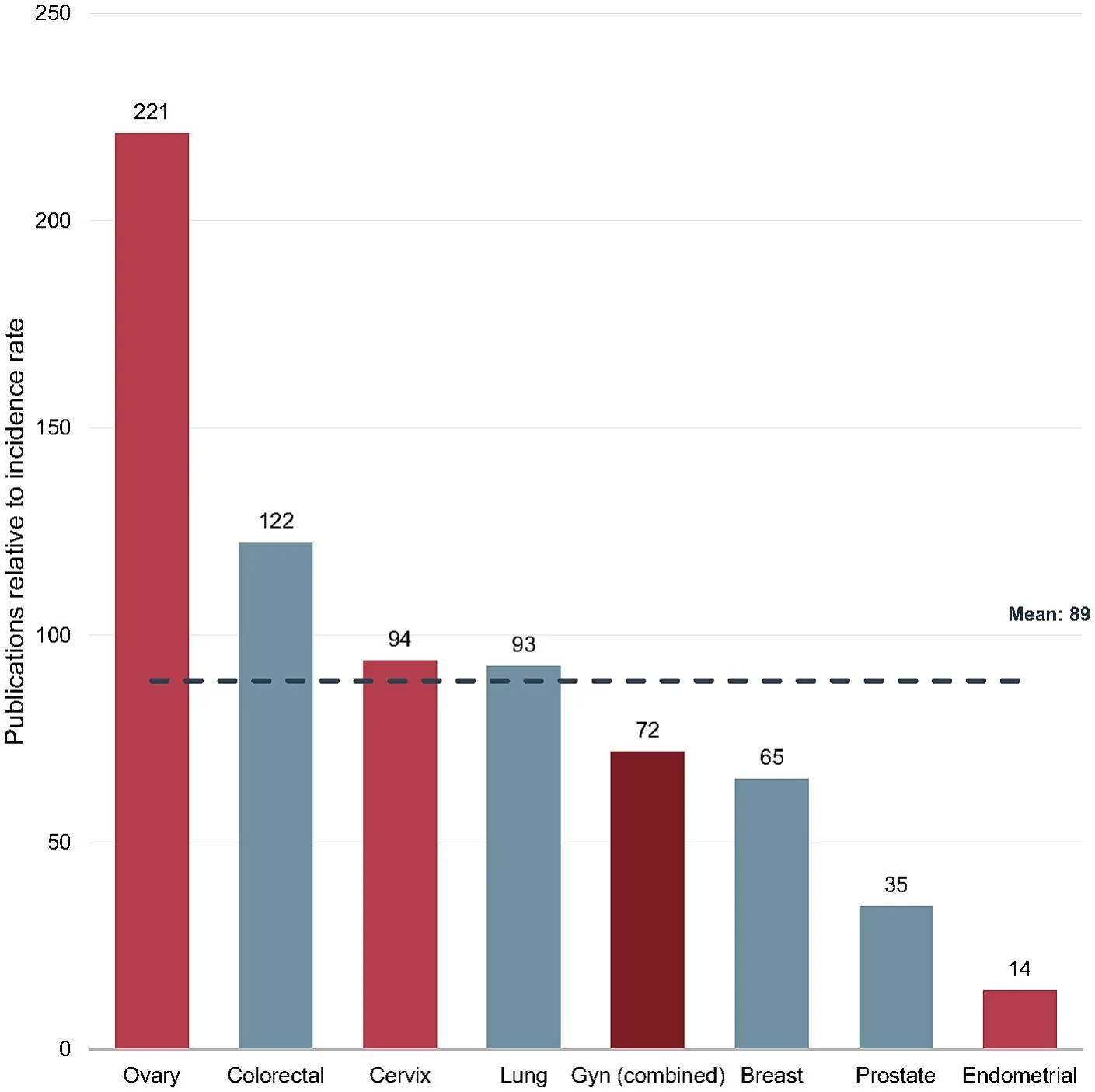

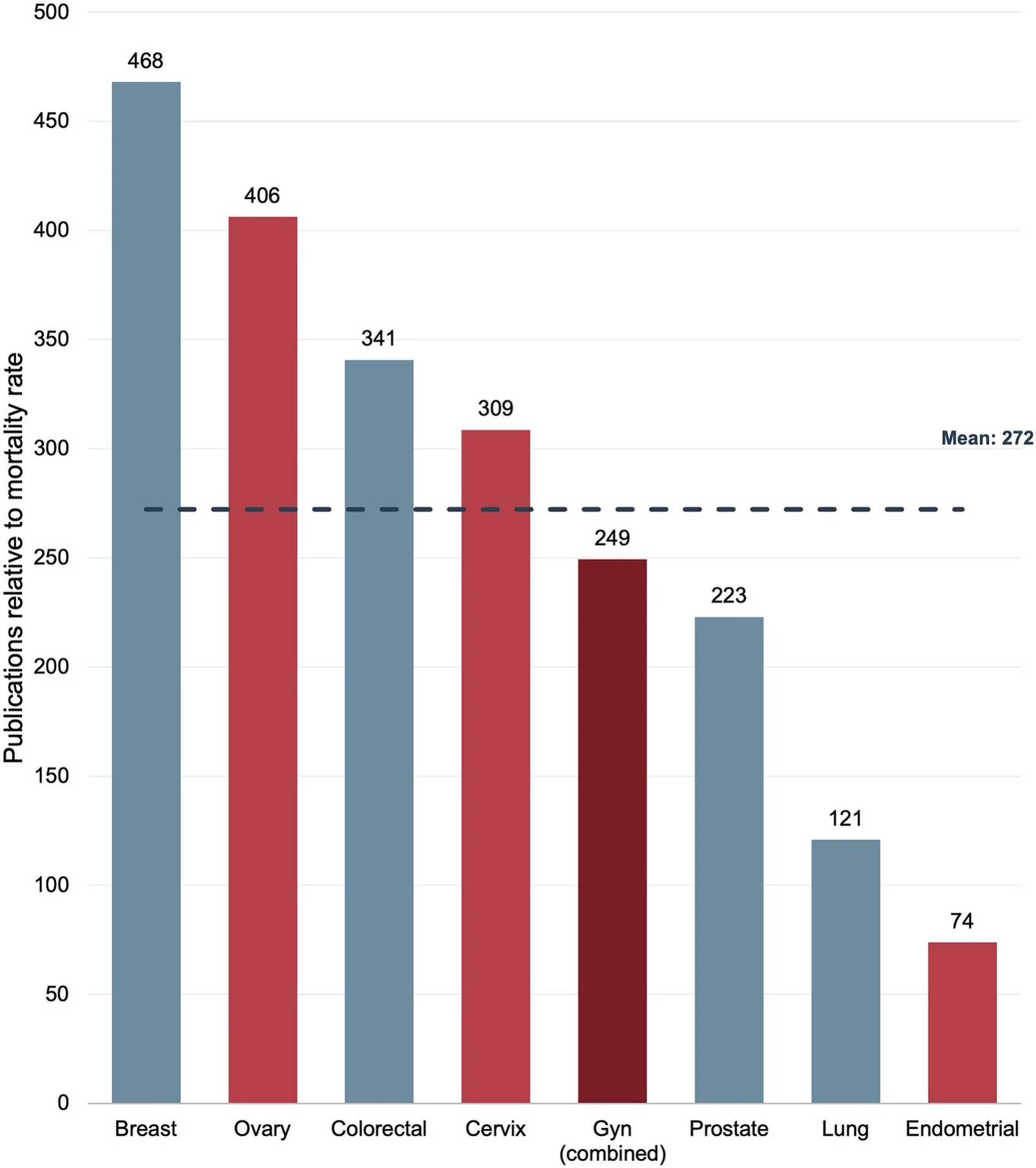

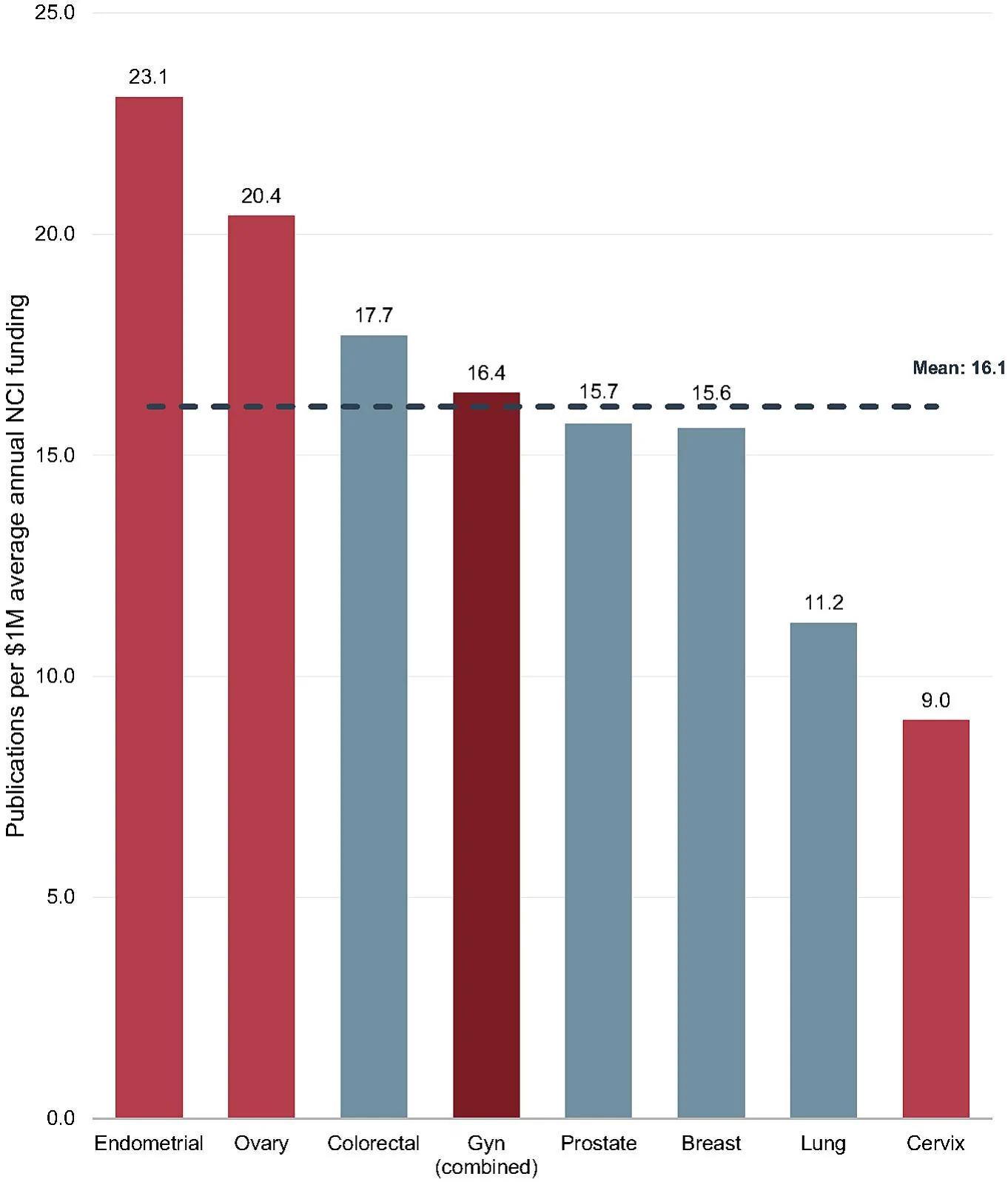
A. Number of basic and translational science publications per case per 100,000. B. Number of basic and translational science publications per death per 100,000. C. Number of basic and translational science publications per $1 million NCI funding. Colors represent cancer category (red: gynecologic cancer, blue: comparison cancers). (For interpretation of the references to color in this figure legend, the reader is referred to the web version of this article.)

Over the study period, the number of publications increased substantially across all cancer types. Increases in average number of publications per year were highest for colorectal (462% increase), cervical (445% increase) and endometrial cancer (346% increase, Supplemental Fig. 1).

## 4 Discussion

Our present study demonstrates that NCI funding relative to disease burden varies considerably among gynecologic cancers, and that endometrial cancer, despite receiving the lowest funding relative to incidence and mortality, was the most productive in basic and translational science output when normalized to funding. Endometrial cancer was found to be particularly productive. Despite receiving only 7.5% of the funding of lung cancer when adjusted for incidence, endometrial cancer generated 23.1 basic and translational science publications per $1 million NCI funding, the highest among all cancer types studied. Additionally, publication output for gynecologic malignancies is increasing at among the highest rates among all cancer types.

Disparities in funding align with prior analyses. Spencer et al. demonstrated that gynecologic cancers were underfunded relative to lethality, as did Kamath et al. (Spencer et al., 2019; Kamath et al., 2019). However, these data were published in 2019 and were based on NCI funding from 2014. Our updated analysis suggests little improvement over time. Recent concerns about stagnating NIH budgets and cuts to federal grants risk further widening this gap (Liu et al., 2025). Advocacy and policy engagement are critical to bridge this funding gap. Ongoing examples include the Society of Gynecologic Oncology's call to support the creation of an endometrial cancer research program through the National Defense Authorization Act (Society of Gynecologic Oncology, n.d.).

Our publication findings were also consistent with those from Sinha et al., in that we too demonstrated lower funding compared to incidence and mortality (Sinha et al., 2023). However, while their research demonstrated that gynecologic malignancies had lower rates of publication when adjusted for lethality, our study found that this varied significantly by cancer type and method of normalization. For example, endometrial cancer had few basic and translational publications when adjusted by incidence and mortality. However, when adjusted for funding, endometrial cancer outpaced other cancers in publication of basic and translational science. These discrepancies may be due to differing inclusion criteria for each study. While Sinha et al. studied all publications, we focused exclusively on basic and translational science. As clinical trials may be more likely to be funded by industry or alternative sources of funding, the strict inclusion of basic and translational science articles may more closely reflect the effects of disparate NCI funding.

Endometrial cancer had the greatest increase in incidence of any cancer studied, but the lowest amount of NCI funding compared to incidence or mortality. Despite decreasing mortality rates for endometrial cancer between 1969 and 1997, these rates have significantly increased since 2001 (Somasegar et al., 2023). Significant racial disparities exist as well, particularly in the mortality among non-Hispanic Black patients with uterine cancer (Eakin et al., 2023). Advances in treatments and early diagnosis rely heavily on basic and translational science output, which has not been equitably funded by the US government (Baker-Rand and Kitson, 2024; Banerjee and Hasson, 2024). Continued efforts to invest in endometrial cancer research are particularly necessary to combat this rapidly increasing mortality rate.

Endometrial cancer had both the highest number of publications per federal dollar spent and one of the greatest rates of publication output increases over the study period. Despite declining NCI funding, increases in publication output for cervical (445%) and endometrial cancers (346%) were second only to colorectal cancer. This suggests that further increased federal investment may continue to yield outsized returns.

Our study was limited by its restriction to high-impact English-language publications, as well as deriving incidence, mortality, and funding data from US-based datasets. Given our reliance on US-based funding and incidence data, findings may not generalize to healthcare systems with different funding structures or epidemiologic profiles (McIntosh et al., 2023). Our findings may also change if pharmaceutical or private funding were included rather than focusing on NCI funding. Additionally, by excluding clinical trials, our findings reflect research output for basic and translational science only, and may not extend to clinical research productivity. Furthermore, publication count is an imperfect surrogate for research impact. A single high-quality discovery may generate more significant scientific and clinical advances than a larger number of more incremental studies. Cross-cancer discoveries that may confer disproportionate benefit to gynecologic cancers (for example, advances in HPV study) would not be adequately captured by our methods. As such, our findings should be interpreted as reflecting research output productivity rather than quality. Lastly, we used Google Scholar h-index as our measure of publication in high-impact journals, though there are alternate sources that have been used in prior publications to distinguish among top journals (Martins and Wasif, 2023). Nevertheless, this study was strengthened by its use of multiple databases to compare basic and translational research output normalized to incidence, mortality, and federal funding. To our knowledge, it is the first such study to do so.

In conclusion, funding relative to disease burden varies considerably among gynecologic cancers, and endometrial cancer in particular remains underfunded despite rising incidence and mortality. Despite this, ovarian and endometrial cancers demonstrated exceptional publication productivity per dollar invested. Specifically, endometrial cancer received the lowest NCI funding relative to incidence and mortality of any cancer studied, yet produced the most basic and translational science publications per NCI dollar. Increased and equitable federal funding support will be critical to further prevention, detection, and treatment advancements for patients with gynecologic malignancies.

## Declaration of generative AI and AI-assisted technologies in the manuscript preparation process.

During the preparation of this work, the author used Generative AI (Claude, Anthropic) in order to assist with manuscript preparation (submission preparation, cross referencing data to ensure consistency). After using this tool/service, the author reviewed and edited the content as needed and takes full responsibility for the content of the published article.

## CRediT authorship contribution statement

**Michael T. Richardson:** Writing – review & editing, Writing – original draft, Visualization, Validation, Supervision, Software, Resources, Project administration, Methodology, Investigation, Funding acquisition, Formal analysis, Data curation, Conceptualization. **Anne Gallivan:** Writing – review & editing, Writing – original draft, Investigation, Data curation. **Lily Zello:** Writing – review & editing, Investigation, Data curation. **Miles Morrow:** Writing – review & editing, Investigation, Data curation. **Daniel S. Kapp:** Writing – review & editing, Methodology, Conceptualization. **John K. Chan:** Writing – review & editing, Conceptualization. **Melissa Herbst-Kralovetz:** Writing – review & editing, Methodology, Investigation, Conceptualization. **Dana M. Chase:** Writing – review & editing, Writing – original draft, Visualization, Supervision, Software, Resources, Project administration, Methodology, Data curation, Conceptualization.

## Funding

Dr. John Chan's research is supported by the Denis Cobb Hale Chair Fund, The Fisher Family Fund, and Angela Wang Johnson Fund.

## Declaration of competing interest

The authors declare that they have no known competing financial interests or personal relationships that could have appeared to influence the work reported in this paper.

## Appendix A Supplementary data

Supplementary material: Supplementary Figure 1. Increase in basic and translational science publication output (%).
