# Supplementary material for: Underfunded, Overperforming: Endometrial cancer leads basic and translational science publications per NCI dollar
Source: Gynecol Oncol Rep. 2026 Nov 3;68:102244. doi: 10.1016/j.gore.2026.102244 (PMC13769480; doi:10.1016/j.gore.2026.102244)
Supplement: Supplementary file 1 [file mmc1.docx]

**Supplementary Figure 1. Increase in basic and translational science publication output (%)**

Percent change (%)

|  | Increase (%) | Mean: 276.1 |
| --- | --- | --- |
| Colorectal | 462.1 | 276.1 |
| Cervix | 444.8 | 276.1 |
| Endometrial | 345.7 | 276.1 |
| Lung | 280.3 | 276.1 |
| Gyn (combined) | 244.4 | 276.1 |
| Ovary | 193.1 | 276.1 |
| Breast | 172.5 | 276.1 |
| Prostate | 66.1 | 276.1 |

Figure displays percent change in average annual publications from 2004-2009 compared to 2018-2024

Colors represent cancer category (red: gynecologic cancer, blue: comparison cancers).

**Supplementary Data**

**Journals Included and H index**

Journals were selected based on Google Scholar’s 2024 H-index rankings under the categories *Health and Medical Sciences* and subcategory *Oncology*. The 20 highest-impact journals from these lists were included, along with selected additional general science journals (specifically, the journals *Nature* and *Science*).

Top 20 from Google Scholar *Health and Medical Sciences*:

1) The New England Journal of Medicine 2) The Lancet 3) Cell 4) JAMA 5) Nature Medicine 6) Proceedings of the National Academy of Sciences 7) BMJ 8) International Journal of Molecular Sciences 9) Journal of Clinical Oncology 10) PLoS One 11) Frontiers in Immunology 12) Circulation 13) International Journal of Environmental Research and Public Health 14) The Lancet Oncology 15) Journal of the American College of Cardiology 16) Nutrients 17) Nature Genetics 18) Morbidity and Mortality Weekly Report 19) The Lancet Infectious Diseases 20) Gastroenterology

Top 20 from Google Scholar *Oncology*:

1) Journal of Clinical Oncology 2) The Lancet Oncology 3) Annals of Oncology 4) Molecular Cancer 5) Cancers 6) Cancer Cell 7) Clinical Cancer Research 8) JAMA Oncology 9) Cancer Discovery 10) Cancer Research 11) Nature Reviews Cancer 12) Nature Reviews Clinical Oncology 13) Frontiers in Oncology 14) Journal of Hematology & Oncology 15) Journal for ImmunoTherapy of Cancer 16) Journal of Experimental & Clinical Cancer Research 17) Journal of Thoracic Oncology 18) Oncogene 19) Leukemia 20) Cancer Letters

**Example PubMed search prompt**

(cervical cancer [title] BMJ [journal]) AND (2004:2024[dp]) NOT (Review[Publication Type])
